# Association of e-cadherin gene CDH1 polymorphism -160 C/A with susceptibility to develop vitiligo^⋆^

**DOI:** 10.1016/j.abd.2022.07.003

**Published:** 2023-02-06

**Authors:** David Emmanuel Kubelis-López, Natalia Aranza Zapata-Salazar, Mauricio Andrés Salinas-Santander, Celia Nohemí Sánchez-Domínguez, Jesús Antonio Morlett-Chávez, Jorge Ocampo-Candiani

**Affiliations:** a Dermatology Department, Hospital Universitario “Dr. José Eleuterio González”, Universidad Autónoma de Nuevo León, Monterrey, Nuevo León, Mexico; b Research Department, Faculty of Medicine Saltillo Unit, Universidad Autónoma de Coahuila, Saltilllo, Coahuila, Mexico; c Department of Biochemistry and Molecular Medicine, Faculty of Medicine “Dr. José Eleuterio González”, Universidad Autónoma de Nuevo León, Monterrey, Nuevo León, Mexico

Dear Editor,

Vitiligo is the most frequent disorder of acquired depigmentation, characterized by achromic patches due to the loss of melanocytes.^1^, ^2^ Its pathogenesis is not completely understood and is believed to be multifactorial.^3^ The melanocytorrhagy theory is based on the observation that mechanical friction leads to melanocyte detachment and transepidermal loss, suggesting that an impaired melanocyte adhesion may be the first step in the development of vitiligo.^2^, ^4^, ^5^ E-cadherin is a Ca^2+^ dependent transmembrane protein and a key adhesion molecule mediating melanocyte-keratinocyte interactions.^3^ The keratinocytes in vitiligo lesions have a weaker expression of e-cadherin and Discoidin Domain Receptor tyrosine kinase 1 (DDR1), another molecule of cell-cell adhesion.^4^ Also, an abnormal distribution of e-cadherin is observed in the normal skin of vitiligo patients, reinforcing the adhesion theory.^4^ There is scarce information on polymorphisms of the e-cadherin gene CDH1 in vitiligo, eight polymorphisms have been previously studied and only the rs10431924 polymorphism showed an association with vitiligo.^2^ Tarlé et al., observed a positive association with T-allele of rs10431924 polymorphism and vitiligo, particularly when accompanied by autoimmune comorbidities in a Brazilian population.^2^ Meanwhile, Almasi-Nasrabadi et al. revealed an association between the CC genotype of rs10431924 and vitiligo in an Iranian population.^1^

This case-control study was conducted aiming to investigate the association between two single nucleotide polymorphisms (SNP) of CDH1 and susceptibility to develop vitiligo in a Mexican population: -347 G→GA (rs5030625) and -160 C/A (rs16260). Both SNP studied are in the promoter region of the CDH1 gene which means that these polymorphisms may lead to over or under-expression of the e-cadherin and thus could have a correlation with the pathogenesis of vitiligo.^6^, ^7^ Patients with a clinical diagnosis of vitiligo and healthy controls without a family history of vitiligo were recruited at the Dermatology Department of the University Hospital “Dr. José Eleuterio González” in Monterrey, México. A venous blood sample was extracted from all subjects for genomic DNA isolation using the salting-out method with a final DNA concentration of 0.1‒1.0 μg/μL. The allele frequency of the CDH1 rs5030625 and rs16260 polymorphisms were characterized by polymerase chain reaction-restriction fragment length polymorphism (PCR-RFLP) using an MJ Mini PTC1148 thermal cycler (Bio-Rad, Hercules; CA, USA). The primers for CDH1 rs5030625 (5’-GCCCCGACTTGTCTCTCTAC-3’ and 5’-GGCCACAGCCAATCAGCA-3’) and CDH1 rs16260 (5’-TGATCCCAGGTCTTAGTGAG-3’ and 5’-AGTCTGAACTGACTTCCGCA-3’) were obtained from IDT (Coralville; IA, USA). According to previously published protocols, the enzymes BanII and BstEII (New England Biolabs; MA, USA) were respectively used in the restriction analysis.^6^, ^8^ All digested products were analyzed by electrophoresis in a 2.5% agarose gel stained with ethidium bromide and visualized in a UVP model 2 UV High-Performance Transilluminator (Upland; CA, USA).

The sample size was calculated considering the vitiligo prevalence in México (4%) and a statistical power of 97.5% (Z = 1.96) resulting in a minimum sample of 114 subjects.^9^ Statistical analysis was performed using IBM SPSS Statistics for Windows version 21.0 (IBM Corp; NY, USA) and Epi Info™ software for Windows version 7 (CDC, USA). A Hardy-Weinberg equilibrium test was obtained for the alleles using a goodness-of-fit test, whereas the genotypic dependence between patients and control subjects was determined with a χ^2^ test. The odd ratios were calculated from 2 × 2 contingency tables. A p < 0.05 was considered significant after the Bonferroni correction.

A total of 116 vitiligo subjects (49 males and 67 females) and 121 controls (45 males and 76 females) were recruited. Demographic characteristics are shown in Table 1. The most frequent type of vitiligo was generalized (n = 97, 83.6%). Frequencies of CDH1 rs5030625 genotypes of the cases and controls are observed in Table 2, the most common genotype was GA/G (cases n = 92, 79.3%; controls n = 102, 84.3%), followed by GA/GA genotype (cases n = 13, 11.2%; controls n = 12, 9.9%) and finally GG genotype (cases n = 11, 9.5%; controls n = 7, 5.8%). After statistical analysis, no association was observed between CDH1 rs5030625 genotypes and vitiligo (p = 0.512). Frequencies of CDH1 rs16260 genotypes are shown in Table 3. In the vitiligo subjects, the AA genotype predominated (n = 70, 60.4%) followed by the CA genotype (n = 39, 33.6%), and lastly CC genotype (n = 7, 6%). In the control group, the AA genotype was the most frequent (n = 54, 47.1%), followed by the CA genotype (n = 54, 44.6%), and the less frequent was the CC genotype (n = 10, 8.3%). Statistical analysis found no association between CDH1 rs16260 genotypes and vitiligo (p = 0.124) but a sub-analysis of a genetic model comparing the AA genotype to CA/CC genotypes showed an association between the risk of developing vitiligo and AA genotype (p = 0.041, OR = 1.709, 95% CI 1.020‒2.861) (Table 3).

**Table 1:** Demographic characteristics of cases and controls.

Variable		Cases (n = 116), n (%)	Controls (n = 121), n (%)
Sex	Male	49 (42.2%)	45 (37.2%)
	Female	67 (57.8%)	76 (62.8%)
Age (years) - mean ± standart deviation		40.15 ± 12.8	31.9 ± 13.8
Vitiligo	Common	97 (83.6%)	NA
	Acrofacial	6 (5.2%)	NA
	Mucosal	1 (0.9%)	NA
	Focal	12 (10.3%)	NA

**Table 2:** Frequency of CDH1 rs5030625 genotypes.

CDH1 rs5030625	Cases (n = 116), n (%)	Controls (n = 121), n (%)	χ*^2^*	p
GG	11 (9.5%)	7 (5.8%)	1.339	0.512
GA/G	92 (79.3%)	102 (84.3%)		
GA/GA	13 (11.2%)	12 (9.9%)

**Table 3:** Frequency of CDH1 rs16260 genotypes.

CDH1 rs16260	Cases (n = 116), n (%)	Controls (n = 121), n (%)	χ^2^	p
AA	70 (60.4%)	57 (47.1%)	4.176	0.1247
CA	39 (33.6%)	54 (44.6%)		
CC	7 (6%)	10 (8.3%)		

CDH1 rs16260	Cases (n = 116), n (%)	Controls (n = 121), n (%)	χ^2^	p	OR	95% CI
AA	70 (60.3%)	57 (47.1%)	4.17	**0.041**	1.709	1.020‒2.861
CA/CC	46 (39.7%)	64 (52.9%)	4.17	**0.041**	0.585	0.349‒0.980

OR, Odds Ratio; CI, Confidence Interval.

Bold are the values that were statistically significant.

This is the first study of CDH1 rs503062 and rs16260 polymorphisms in vitiligo; an association between the AA genotype of CDH1 rs16260 and the risk of developing vitiligo was observed when compared to CC/CA genotypes. This study reinforces the e-cadherin role in the development of vitiligo. Further studies of rs16260 CDH1 polymorphism are necessary to confirm these findings.

## Ethics

This study was conducted in accordance with the Declaration of Helsinki, and it was approved by the University Hospital “Dr. José Eleuterio González” research and ethical committee with registry number DE20-00013.

## Financial support

None declared.

## Authors’ contributions

David Emmanuel Kubelis-López: Study concept and design; data collection, analysis, and interpretation of data; writing of the manuscript; critical review of the literature and final approval of the final version of the manuscript.

Natalia Aranza Zapata-Salazar: Study concept and design; data collection, analysis, and interpretation of data; writing of the manuscript; critical review of the literature and final approval of the final version of the manuscript.

Mauricio Andrés Salinas-Santander: Study concept and design; research guidance, data collection, analysis, and interpretation of data; writing of the manuscript; critical review of the literature and final approval of the final version of the manuscript.

Celia Nohemí Sánchez-Domínguez: Study concept and design; research guidance; data collection, analysis, and interpretation of data; writing of the manuscript; critical review of the literature and final approval of the final version of the manuscript.

Jesús Antonio Morlett-Chávez: Data collection, analysis, and interpretation of data; review of the manuscript; critical review of literature and final approval of the final version of the manuscript.

Jorge Ocampo-Candiani: Study concept and design; research guidance; data collection, analysis, and interpretation of data; writing of the manuscript; critical review of the literature and final approval of the final version of the manuscript.

## Conflicts of interest

None declared.
